# Lower rates of ART initiation and decreased retention among ART-naïve patients who consume alcohol enrolling in HIV care and treatment programs in Kenya and Uganda

**DOI:** 10.1371/journal.pone.0240654

**Published:** 2020-10-23

**Authors:** Ioannis Patsis, Suzanne Goodrich, Constantin T. Yiannoutsos, Steven A. Brown, Beverly S. Musick, Lameck Diero, Jayne L. Kulzer, Mwembesa Bosco Bwana, Patrick Oyaro, Kara K. Wools-Kaloustian

**Affiliations:** 1 Department of Hygiene and Epidemiology, School of Medicine, National and Kapodistrian University of Athens, Athens, Greece; 2 Division of Infectious Diseases, Department of Medicine, Indiana University School of Medicine, Indianapolis, Indiana, United States of America; 3 Department of Biostatistics, Indiana University Fairbanks School of Public Health, Indianapolis, Indiana, United States of America; 4 Department of Biostatistics, Indiana University School of Medicine, Indianapolis, Indiana, United States of America; 5 School of Medicine, College of Health Sciences, Moi University, Eldoret, Kenya; 6 Department of Medicine, University of California San Francisco, San Francisco, California, United States of America; 7 Department of Medicine, Mbarara University of Science and Technology, Mbarara, Uganda; 8 Centre for Microbiology Research, Kenya Medical Research (KEMRI), Nairobi, Kenya; University of the Witwatersrand, SOUTH AFRICA

## Abstract

**Objectives:**

Almost 13 million people are estimated to be on antiretroviral therapy in Eastern and Southern Africa, and their disease course and program effectiveness could be significantly affected by the concurrent use of alcohol. Screening for alcohol use may be important to assess the prevalence of alcohol consumption and its impact on patient and programmatic outcomes.

**Methods:**

As part of this observational study, data on patient characteristics and alcohol consumption were collected on a cohort of 765 adult patients enrolling in HIV care in East Africa. Alcohol consumption was assessed with the AUDIT questionnaire at enrollment. Subjects were classified as consuming any alcohol (AUDIT score >0), hazardous drinkers (AUDIT score ≥8) and hyper drinkers (AUDIT score ≥16). The effects of alcohol consumption on retention in care, death and delays in antiretroviral therapy (ART) initiation were assessed through competing risk (Fine & Gray) models.

**Results:**

Of all study participants, 41.6% consumed alcohol, 26.7% were classified as hazardous drinkers, and 16.0% as hyper drinkers. Depending on alcohol consumption classification, men were 3–4 times more likely to consume alcohol compared to women. Hazardous drinkers (median age 32.8 years) and hyper drinkers (32.7 years) were slightly older compared to non-hazardous drinkers (30.7 years) and non-hyper drinkers (30.8 years), (p-values = 0.014 and 0.053 respectively). Median CD4 at enrollment was 330 cells/μl and 16% were classified World Health Organization (WHO) stage 3 or 4. There was no association between alcohol consumption and CD4 count or WHO stage at enrollment. Alcohol consumption was associated with significantly lower probability of ART initiation (adjusted sub-distribution hazard ratio aSHR = 0.77 between alcohol consumers versus non-consumers; p-value = 0.008), and higher patient non-retention in care (aSHR = 1.77, p-value = 0.023).

**Discussion:**

Alcohol consumption is associated with significant delays in ART initiation and reduced retention in care for patients enrolling in HIV care and treatment programs in East Africa. Consequently, interventions that target alcohol consumption may have a significant impact on the HIV care cascade.

## Introduction

In 2017, there were 19.6 million [17.5 million– 22.0 million] people with HIV (PWH) in Eastern and Southern Africa [1]. As the number of patients accessing HIV care has rapidly increased in the past ten years, nearly 12.9 million people [11.4 million- 13.4 million] in Eastern and Southern Africa are now on antiretroviral therapy (ART) [1]. However, retention in care remains a significant challenge [2]. For example, in East Africa only about 69% of patients initiating ART remain in care at the clinic of their initial enrollment after two years[3]. Patients who are disengaged from care have significantly higher mortality [2, 4, 5] and HIV transmission rates compared to those who remain engaged in care [6, 7]. Consequently, the investigation of factors that impact patterns of ART initiation and retention in care for patients enrolling in these programs is of particular significance.

Though the association between alcohol consumption and poor health outcomes in HIV infection is well established [8–10], the impact of alcohol consumption on patients’ initiation of ART and retention in care has yet to be thoroughly examined. Nevertheless, there is some recent evidence that drinking is associated with poor retention in care, in both resource-replete and resource-limited settings [11, 12]. Heavy drinking and frequent binge drinking are associated with worse retention in HIV care in patients observed at seven U.S. HIV clinical sites [11], and findings from a systematic review suggest that low-income and middle-income countries (as classified by the World Bank) face issues similar to those of high-income countries [12]. Despite that, only a few evidence-based interventions specifically target problematic alcohol consumption [12]. In addition, measurement of alcohol exposure and retention measures varies among studies so that generalizability of their findings is an issue. Moreover, adjusting for all possible confounders in such studies is challenging, and causality cannot be inferred [11]. In Africa, 42.6% of the population is estimated to consume alcohol [13]. Thus, the impact of alcohol on the course of HIV infection and the effectiveness of care and treatment programs, which rely on rapid initiation of patients on ART and patient retention into care for their success, may be significantly affected by ambient alcohol consumption patterns, particularly in resource-limited areas with high HIV prevalence like sub-Saharan Africa.

The purpose of this observational study was to determine the prevalence of alcohol consumption in ART-naïve patients initiating HIV care and to assess whether alcohol consumption is associated with time to ART initiation, mortality, and patient retention in care.

## Methods

### Study design

This prospective observational study was approved by the Indiana University Institutional Review Board and the ethical bodies affiliated with each participating site: The Academic Model Providing Access to Healthcare (AMPATH): Moi University College of Health Sciences and MOI Teaching and Referral Hospital’s Institutional Research and Ethics Committee; Family AIDS Care and Education Services (FACES): Kenya Medical Research Institute/National Ethics Review Committee; Mbarara Immune Suppression Syndrome (ISS) Clinic: Mbarara University of Science & Technology Institutional Review Committee. Participant written informed consent was obtained at the time of enrollment into study.

### Study setting and standard of care

The study took place in five clinics within the East Africa International epidemiology Databases to Evaluate AIDS (EA-IeDEA) consortium. EA-IeDEA is one of seven regional consortia supported by the National Institutes of Health to consolidate, curate and analyze HIV care and treatment data in order to evaluate the outcomes of people living with HIV/AIDS [14]. Study sites included the FACES clinics at Lumumba sub-County Hospital, (Kisumu Kenya) and Suba sub-County Hospital (Sindo, Kenya); two AMPATH clinics based at Moi Teaching and Referral Hospital (Eldoret, Kenya); and the Mbarara ISS Clinic (Mbarara, Uganda). The sites in Eldoret and Kisumu are primarily urban, while Sindo is rural and Mbarara is semi-urban. Each clinic provides comprehensive HIV care including CD4 and HIV viral load testing, provision of ART, diagnosis and treatment of opportunistic infections, as well as management of common non-communicable diseases.

Individuals enrolling in care have blood drawn for a CD4 count and undergo World Health Organization (WHO) disease staging by a clinician [15]. Patients started on trimethoprim-sulfa prophylaxis, if no contraindication existed. Eligibility criteria for ART was assessed as per WHO guidelines during the study period, hence individuals were eligible for ART if they had a CD4 count below 350 cells/μl and/or a WHO stage of >2 [16].

Patients not meeting ART eligibility criteria returned to clinic at a frequency determined by the clinic, but usually at a maximum of every six months, for care and continued monitoring of their CD4 count for ART eligibility. For patients initiating ART, CD4 counts were repeated at 6–12 months depending on the clinics testing strategy. Routine viral load testing was not always available during the study period.

### Study population

Participants were recruited from January 25, 2013 to June 25, 2014. Participants met eligibility criteria for inclusion in this study if they were at least 18 years of age, newly enrolling in HIV-care at one of the five participating clinics, and ART-naïve. Potential participants were excluded if they did not meet any of the above criteria or were unable or unwilling to provide consent for the study. All participants were educated about the study and provided written consent prior to enrollment.

The Alcohol Use Disorder Identification Test (AUDIT) questionnaire, which has been validated in a number of primary care medical settings, was used by a trained research assistant to collect patient’s alcohol use data over the past one year [17–20]. The AUDIT version utilized is comprised of 10 questions covering three domains: hazardous alcohol use, dependence symptoms, and harmful alcohol use. The total AUDIT score is calculated by summing the values from each question (range 0–4). RAs attempted to minimize under-reporting of a participant's alcohol use by asking in-depth questions about the types of alcohol consumed, the volume (facilitated by showing participants a large collection of glasses and bottles in varying shapes and sizes) and frequency of consumption. Using a spreadsheet with the known alcohol content of commercially available drinks and estimates of the alcohol content of local (non-commercial) brews [21], calculations were made to determine the number of standard drinks (one drink defined as 10 grams of alcohol) each participant consumed.

Additional information was collected as part of routine care including demographics (e.g., age, gender, civil status, HIV disclosure), and clinical data (e.g., CD4 count and WHO disease stage at enrollment). Locator information including addresses and telephone numbers were obtained as part of clinic enrollment and then verified and updated as needed by study personnel. Participants who failed to return for a scheduled visit for more than two months were traced by trained outreach personnel. The first attempt was made by telephone, and, if unsuccessful, community tracing was attempted. Once the participant or a reliable close informant was found, vital status and engagement in care were documented.

### Statistical methods

#### Definition of the outcomes of interest

The three outcomes of interest were time from enrollment in care to ART initiation, mortality, and retention in care. Retention in care was measured by rates of patients actually disengaged from care as ascertained by tracing patients in the community who failed to return to clinic within two months from their last clinic visit to ensure they had not died or were receiving care elsewhere [22, 23]. In this manner, we were able to estimate patient retention in care from the perspective of the patient rather than that of the program. On the basis of tracing and community outreach, participants were classified as silent transfers (undocumented transfer to another care facility), or as having relocated, deceased, disengaged, or missing (untraceable). Within these analyses, censoring occurred at the date patients were traced or, if not located, at the last clinic visit for patients who were determined to be silent transfers and participants who had relocated, because these outcomes were not considered adverse outcomes from a programmatic perspective and were classified as being retained in care. By contrast, patients who were untraceable (missing) or who were found to have disengaged from care were considered as having adverse programmatic outcomes and so were classified as not having been retained in care.

#### Predictors used in the analyses

Utilizing WHO criteria binary categories based on AUDIT scores were developed: non-alcohol consumers (AUDIT = 0) versus alcohol consumers (AUDIT > 0); people engaged in hazardous drinking (AUDIT ≥ 8) versus not (AUDIT < 8); and people engaged in high alcohol consumption (combined categories of harmful drinking and risk for dependence; AUDIT ≥16) versus not (AUDIT <16) [13].

The covariates of interest included gender, age (18–24, 25–34, 35–44, 45+), civil status (legally married versus not), CD4 cell count at enrollment (<50, 50–99, 100–199, 200–349, 350–499, 500+), WHO stage (1/ 2 or 3/ 4) and HIV status disclosure (whether the participant has disclosed HIV carrier status to anyone) at the time of enrollment. The five participating clinics were grouped under their parent programs: FACES, AMPATH and Mbarara. Missing values for CD4 cell count, WHO stage, civil status, and HIV disclosure multiple imputation was utilized using iterative chained equations (ICE) under the assumption that the missing values were missing at random (MAR) [24].

#### Statistical modeling

Descriptive statistics were used to calculate the prevalence of alcohol consumption. The association between age, CD4 count at enrollment and alcohol consumption was examined by the Mann-Whitney test. The Pearson chi-square test was used to examine the possible association between categorical variables like gender, WHO stage, age and the AUDIT alcohol consumption categories.

To examine the impact of alcohol use on delays in ART initiation, retention in care and mortality, survival analysis techniques were used. Study participants were at risk of experiencing more than one outcome. However, observation of one outcome such as death or non-retention, prior to another, such as, for example, ART initiation, would preclude observing the other outcome. As such, all events other than the event of interest were considered competing risks [25]. To assess the impact of alcohol consumption on each event of interest, in the presence of all competing events and other explanatory factors, we used the method of Fine and Gray in the analysis [25]. We performed three separate analyses, each investigating the possible effect of different levels of alcohol consumption (i.e., any alcohol use, hazardous and higher or harmful alcohol consumption, the exposure) on the likelihood (sub-distribution hazard) of each of the three events of interest (ART initiation, death, and non-retention in care). In all cases we adjusted for all relevant patient-level characteristics. With regard to the event of ART initiation, the competing risks were non-retention in care and death, whereas for mortality, the corresponding competing risks were non-retention and ART initiation. The competing risk for the event of non-retention was death.

Time-to-event analyses were conducted under the assumption of independent censoring. This means that the failure rate for subjects within a subgroup (e.g., people engaged in hazardous alcohol consumption) who had relocated or silently transferred is assumed to be equal to the failure rate for subjects in that subgroup who remained on observation and had not experienced any event until that time-point [26]. Associations with corresponding p-values <5% were designated as statistically significant, and as possibly significant if between 5% and 10%. Data analyses were conducted using the statistical program Stata version 13 (StataCorp, College Station, Texas).

## Results

### Participant characteristics

A total of 765 patients with median age of 31.2 years at enrollment participated in the study (Table 1); more than half were women (61%). At enrollment, the median CD4 count was 330 cells/μl (IQR: 137–513) and 84% (n = 591) of study participants with a known WHO stage had a WHO stage 1 or 2 disease. About two thirds of the participants with a known civil status were legally married (66%, n = 361) and just over half of those with a known disclosure status had disclosed their HIV status (56%, n = 269). As classified by AUDIT scores, 41.6% of study participants consumed alcohol, with 26.7% scoring at least in the hazardous range and 16.0% in the harmful drinking range or being at risk for dependency.

**Table 1:** Characteristics of the study cohort.

	Overall (n:765)	no alcohol consumption	alcohol consumption^1^	non-hazardous alcohol consumption	Hazardous alcohol consumption^2^	Non-harmful drinking	Harmful drinking^3^
**Overall (n:765)**		447 (58.4)	318 (41.6)	561(73.3)	204 (26.7)	643 (84.1)	122 (16.0)
IeDEA program p-value*		<0.001		0.062		<0.001	
AMPATH	264 (34.5)	166 (37.1)	98 (30.8)	184 (32.8)	80 (39.2)	209 (32.5)	55 (45.1)
Mbarara	264 (34.5)	130 (29.1)	134 (42.1)	207 (36.9)	57 (27.9)	245 (38.1)	19 (15.6)
FACES	237 (31.0)	151 (33.8)	86 (27.0)	170 (30.3)	67 (32.8)	189 (23.4)	48 (39.3)
**Total**	765	447	318	561	204	643	122
Gender [N (%)] p-value*		<0.001		<0.001		<0.001	
Male	295 (38.6)	120 (26.9)	175 (55.0)	164 (29.2)	131 (64.2)	213 (33.1)	82 (67.2)
Female	470 (61.4)	327 (73.2)	143 (45.0)	397 (70.8)	73 (35.8)	430 (66.9)	40 (32.8)
**Total**	765	447	318	561	204	643	122
Age [median (IQR)] p-value**		NS		0.014		0.053	
	31.2 (26.1, 39.5)	30.8 (25.8, 39.8)	32.0 (26.9, 39.4)	30.7 (25.8, 38.9)	32.8 (27.6, 40.5)	30.8 (25.8, 39.4)	32.7 (27.8, 40.7)
Age (categorical) [N (%)] p-value*		NS		NS		NS	
18–24	147 (19.2)	97 (21.7)	50 (15.7)	120 (21.4)	27 (13.2)	134 (20.8)	13 (10.7)
25–34	338 (44.2)	186 (41.6)	152 (47.8)	243 (43.3)	95 (46.6)	279 (43.4)	59 (48.4)
35–44	182 (23.8)	107 (23.9)	75 (23.6)	128 (22.8)	54 (26.5)	147 (22.9)	35 (28.7)
>44	98 (12.8)	57 (12.8)	41 (12.9)	70 (12.5)	28 (13.7)	83 (12.9)	15 (12.3)
WHO stage at enrollment [N (%)] p-value*		NS		NS		NS	
1–2	591 (84.0)	341 (84.2)	250 (83.6)	437 (85.2)	154 (80.6)	500 (84.6)	91 (80.5)
3–4	113 (16.1)	64 (15.8)	49 (16.4)	76 (14.8)	37 (19.4)	91 (15.4)	22 (19.5)
**Total (non-missing)**	704	405	299	513	191	591	113
Missing	61 (8.0)	42 (9.4)	19 (6.0)	48 (8.6)	13 (6.4)	52 (8.1)	9 (7.4)
CD4 at enrollment [median (IQR)] p-value**		NS		NS		NS	
	330 (137, 513)	316 (148, 534)	342 (127, 487)	325 (150, 525)	342 (115, 479)	329 (141, 515.5)	330 (124, 469)
Missing [N (%)]	197 (25.8)	130 (29.1)	67 (21.1)	142 (25.3)	55 (27.0)	159 (24.7)	38 (31.1)
**CD4 count (categorical) [N (%)]**							
[0–49]	67 (11.8)	37 (11.7)	30 (12.0)	47 (11.2)	20 (13.4)	56 (11.6)	11 (13.1)
[50–99]	45 (7.9)	23 (7.3)	22 (8.8)	32 (7.6)	13 (8.7)	38 (7.9)	7 (8.3)
[100–249]	119 (21.0)	73 (23.0)	46 (18.3)	92 (22.0)	27 (18.1)	102 (21.1)	17 (20.2)
[250–349]	70 (12.3)	39 (12.3)	31 (12.4)	53 (12.7)	17 (11.4)	59 (12.2)	11 (13.1)
[350–499]	117 (20.6)	57 (18.0)	60 (23.9)	79 (18.9)	38 (22.5)	97 (20.0)	20 (23.8)
≥500	150 (26.4)	88 (27.8)	62 (24.7)	116 (27.7)	34 (22.8)	132 (27.3)	18 (21.4)
**Total**	568	317	251	419	149	484	84
Civil status [N (%)] p-value*		NS		NS		NS	
Not married	189 (34.4)	101 (32.8)	88 (36.4)	139 (34.3)	50 (34.5)	165 (35.2)	24 (29.6)
Married	361 (65.6)	207 (67.2)	154 (63.6)	266 (65.7)	95 (65.5)	304 (64.8)	57 (70.4)
**Total (non-missing)**	550	308	242	405	145	469	81
Missing	215 (28.1)	139 (31.1)	76 (23.9)	156 (27.8)	59 (28.9)	174 (27.1)	41 (33.6)
HIV status disclosure [N (%)] p-value*		NS		NS		NS	
Not disclosed	208 (43.6)	135 (44.9)	73 (41.5)	148 (43.8)	60 (43.2)	165 (43.4)	43 (44.3)
Disclosed	269 (56.4)	166 (55.2)	103 (58.5)	190 (56.2)	79 (56.8)	215 (56.6)	54 (55.7)
**Total (non-missing)**	477	301	176	338	139	380	97
Missing	288 (37.6)	146 (32.7)	142 (44.7)	223 (39.8)	65 (31.9)	263 (40.9)	25 (20.5)

^**1**^AUDIT score of >0

^**2**^AUDIT score of ≥8

^**3**^AUDIT score of ≥16

NS: non-significant

*Chi-square test

**Mann-Whitney test.

Men were more likely than women to consume alcohol regardless of the categorization of alcohol consumption (Table 1). In fact, the odds of belonging to the higher alcohol consumption category were between 3-fold and 4-fold higher for men compared to women. Older participants were more likely to belong to higher alcohol consumption categories when compared to younger participants. However, no age-by-gender interaction was identified. There was no association between enrollment CD4 count, WHO stage, or HIV disclosure status and alcohol consumption (Table 1).

Initially, 25% (n = 190) of participants were identified as lost to program. After tracing, 38% were found to be silent (unreported) transfers, 29% disengaged from care, 7% deceased, 12% relocated and 13% untraceable (missing). During the study period, 28 (3.7%) participants died.

### Competing-risk analysis of the events of interest

Consumption of alcohol was strongly associated with a higher (sub-distribution) hazard of not being retained in care in a competing-risk analysis with death as the competing event. Adjusted for all relevant factors, patients who consumed any amount of alcohol had a 77% higher hazard of being non-retained in care (adjusted sub-distribution hazard rate–aSHR– 1.77, p-value = 0.023) compared to those not consuming alcohol. Given the low rates of non-retention (10.6%, Table 2), this is approximately equal 77% higher rate of non-retention. Other alcohol exposure classifications (i.e., hazardous versus non-hazardous and harmful versus non-harmful alcohol consumption) were not associated with patient retention (Table 3).

**Table 2:** Events used in the survival analyses.

Main event	Competing event(s)	Alcohol consumption			Hazardous drinking			Harmful drinking		
		No	Yes	Total	No	Yes	Total	No	Yes	Total
Total		447	318	765	561	204	765	643	122	765
***Non-retention*** *[N(%)]*		41 (9.2)	40 (12.6)	81 (10.6)	55 (9.8)	26 (12.8)	81 (10.6)	63 (9.8)	18 (14.8)	81 (10.6)
	Death *[N(%)]*	14 (3.1)	14 (4.4)	28 (3.7)	16 (2.9)	12 (5.9)	28 (3.7)	22 (3.4)	6 (4.9)	28 (3.7)
	Censored *[N(%)]*	392 (87.7)	264 (83.0)	656 (85.8)	490 (87.3)	166 (81.4)	656 (85.8)	558 (86.8)	98 (80.3)	656 (85.8)
***Death*** *[N(%)]*		14 (3.1)	14 (4.4)	28 (3.7)	16 (2.9)	12 (5.9)	28 (3.7)	22 (3.4)	6 (4.9)	28 (3.7)
	Non-retention *[N(%)]*	41 (9.2)	40 (12.6)	81 (10.6)	55 (9.8)	26 (12.8)	81 (10.6)	63 (9.8)	18 (14.8)	81 (10.6)
	Censored *[N(%)]*	392 (87.7)	264 (83.0)	656 (85.8)	490 (87.3)	166 (81.4)	656 (85.8)	558 (86.8)	98 (80.3)	656 (85.8)
***ART initiation*** *[N(%)]*		313 (70.0)	208 (65.4)	521 (68.1)	386 (68.8)	135 (66.2)	521 (68.1)	441 (68.6)	80 (65.6)	521 (68.1)
	Non-retention *[N(%)]*	21 (4.7)	26 (8.2)	47 (6.1)	29 (5.2)	18 (8.8)	47 (6.1)	34 (5.3)	13 (10.7)	47 (6.1)
	Death *[N(%)]*	5 (1.1)	3 (0.9)	8 (1.1)	5 (0.9)	3 (1.5)	8 (1.1)	6 (0.9)	2 (1.6)	8 (1.1)
	Censored *[N(%)]*	108 (24.2)	81 (25.5)	189 (24.7)	141 (25.1)	48 (23.5)	189 (24.7)	162 (25.2)	27 (22.1)	189 (24.7)

**Table 3:** Results of the Fine & Gray competing events survival models, adjusting for age, gender, CD4 count and WHO stage at enrollment, disclosure of HIV status, marital status and site of enrollment; aSHR: adjusted sub-distribution hazard.

		Any alcohol use		Hazardous alcohol consumption		Harmful alcohol consumption	
Main event	Competing risk	aSHR [95% CI]	(p-value)	aSHR [95% CI]	(p-value)	aSHR [95% CI]	(p-value)
*Non-retention*	Death	1.766 [1.083, 2.879]	0.023	1.380 [0.737, 2.389]	0.250	1.480 [0.817, 2.681]	0.195
*Death*	Non-retention	1.164 [0.580, 2.334]	0.669	1.736 [0.799, 3.773]	0.163	1.245 [0.464, 3.345]	0.663
*ART initiation*	Non-retention Death	0.770 [0.636, 0.933]	0.008	0.841 [0.680, 1.040]	0.111	0.814 [0.623, 1.064]	0.132

Consumption of alcohol was also associated with a reduced likelihood (sub-distribution hazard) of ART initiation. In the adjusted analysis, patients who consumed any alcohol were approximately 25% less likely to initiate ART as compared to persons who do not consume alcohol (aSHR: 0.77; p-value = 0.008). No association was observed between the other alcohol consumption classifications and the likelihood of ART initiation (Table 3).

Alcohol consumption, regardless of classification, was not associated with an increased hazard of mortality (Table 3).

## Discussion

This study found a strong association between patient’s alcohol consumption in the past one year and retention in HIV care. Association between higher levels of alcohol consumption and patient retention in care was less clear, suggesting that it may be the consumption of alcohol *per se* which may be associated with patient retention rather than the amount of consumed alcohol. These results are largely consistent with a large U.S.-based study [11]. However, that study, which included more men (82%) who were identified as heavy drinkers (25% vs. 16% in our study), found a stronger association (adjusted OR 0.78) between heavy alcohol consumption and non-retention, something that our study failed to establish. A systematic review also identified the adverse impact of alcohol use on the HIV care cascade (diagnosis, linkage to care and retention) but only 4 out of 53 studies in that review specifically focused on retention in care and only one was set in a low-income country [12].

Our analysis showed that patients who consumed alcohol had a lower likelihood of initiating ART. In a systematic review [12] three out of six studies looking specifically at ART initiation had similar findings, with the others failing to show a significant association between alcohol consumption and delay in ART initiation. Given that alcohol consumption did not have an effect on ART eligibility in our data (analysis not shown), lower rates of ART initiation among patients that consume alcohol may have occurred because these patients may not have met assessment criteria suggesting readiness to start ART and to maintain adherence to the ART regimen. As a result, providers may have been reluctant to initiate patients who consume alcohol on ART, thus delaying the start of therapy. Interestingly, a study from Uganda [27] showed a majority of patients who were previously consuming alcohol and started on ART subsequently abstained from alcohol over three years of follow-up. While providers may perceive that delay in ART initiation is advantageous, ART start may actually improve abstinence to alcohol and allow for the public health benefit of decreasing patients’ viral load.

Alcohol consumption was not associated with higher mortality in our cohort. Mortality was generally a rare event in our study given the short follow-up time (less than one year for most participants) and the fact that the majority of our patients were relatively healthy at enrollment as determined by CD4 count and WHO stage (Table 1). Consequently, with less than 4% of study subjects dying during the study, our study was not powered to detect any potential effects of alcohol consumption on mortality.

We found no association between alcohol consumption and HIV disease severity in patients enrolling in care based on WHO stage and CD4 cell count. These results are consistent with prior studies in which alcohol dependence was associated with lower CD4 counts, but moderate alcohol consumption was not [28–31]. Moreover, given the small proportion of high alcohol consumption in our study, such an effect might be difficult to detect.

Our inability to establish a clear association between the highest levels of alcohol consumption and adverse patient and programmatic outcomes may be due to inaccurate patient reporting despite the significant effort made by our study staff to determine the actual number of standardized drinks consumed. It is possible that some participants attempted to offer more socially acceptable descriptions of their alcohol consumption patterns, which may have resulted in an underestimation of the number of heavy drinkers. In addition, the alcohol content of domestically produced brews varies and thus is difficult to assess. Combined, these limitations in our data collection may account for finding an association of alcohol consumption with decreased retention in care and delays in ART initiation, but not confirming the same findings among those at the highest alcohol consumption classifications.

## Conclusion

Our results show that alcohol consumption, irrespective of amount, is strongly associated both with lower rates of ART initiation and lower patient retention in care (respectively the second and the critical third component of the WHO’s 90-90-90 target [1]). Consequently, its evaluation appears to be a significant target for intervention in HIV care. With a high prevalence of alcohol consumption in sub-Saharan Africa, alcohol use is a factor that programs should consider addressing as they implement and employ universal testing and treatment for all people living with HIV [16].

## References

[1] (UNAIDS) JUNPoHA. 90-90-90: An ambitious treatment target to help end the AIDS epidemic. Geneva: Joint United Nations Programme on HIV/AIDS (UNAIDS), 2014.

[2] Giordano TP, Gifford AL, White AC Jr., Suarez-Almazor ME, Rabeneck L, Hartman C, et al Retention in care: a challenge to survival with HIV infection. Clin Infect Dis. 2007;44(11):1493–9. Epub 2007/05/08. 10.1086/516778 . 17479948

[3] Geng EH, Odeny TA, Lyamuya R, Nakiwogga-Muwanga A, Diero L, Bwana M, et al Retention in Care and Patient-Reported Reasons for Undocumented Transfer or Stopping Care Among HIV-Infected Patients on Antiretroviral Therapy in Eastern Africa: Application of a Sampling-Based Approach. Clin Infect Dis. 2016;62(7):935–44. Epub 2015/12/19. 10.1093/cid/civ1004 26679625 PMC4787603

[4] Mugavero MJ, Lin HY, Willig JH, Westfall AO, Ulett KB, Routman JS, et al Missed visits and mortality among patients establishing initial outpatient HIV treatment. Clin Infect Dis. 2009;48(2):248–56. Epub 2008/12/17. 10.1086/595705 19072715 PMC2737584

[5] Tripathi A, Youmans E, Gibson JJ, Duffus WA. The impact of retention in early HIV medical care on viro-immunological parameters and survival: a statewide study. AIDS Res Hum Retroviruses. 2011;27(7):751–8. Epub 2010/12/15. 10.1089/AID.2010.0268 . 21142607

[6] De Cock KM, Gilks CF, Lo YR, Guerma T. Can antiretroviral therapy eliminate HIV transmission? Lancet. 2009;373(9657):7–9. Epub 2008/11/29. 10.1016/S0140-6736(08)61732-8 . 19038440

[7] Eaton JW, Johnson LF, Salomon JA, Barnighausen T, Bendavid E, Bershteyn A, et al HIV treatment as prevention: systematic comparison of mathematical models of the potential impact of antiretroviral therapy on HIV incidence in South Africa. PLoS Med. 2012;9(7):e1001245 Epub 2012/07/18. 10.1371/journal.pmed.1001245 22802730 PMC3393664

[8] Bagby GJ, Amedee AM, Siggins RW, Molina PE, Nelson S, Veazey RS. Alcohol and HIV Effects on the Immune System. Alcohol Res. 2015;37(2):287–97. Epub 2015/12/24. 26695751 10.35946/arcr.v37.2.12 PMC4590624

[9] Baliunas D, Rehm J, Irving H, Shuper P. Alcohol consumption and risk of incident human immunodeficiency virus infection: a meta-analysis. Int J Public Health. 2010;55(3):159–66. Epub 2009/12/02. 10.1007/s00038-009-0095-x . 19949966

[10] Pandrea I, Happel KI, Amedee AM, Bagby GJ, Nelson S. Alcohol's role in HIV transmission and disease progression. Alcohol Res Health. 2010;33(3):203–18. Epub 2010/01/01. 23584062 PMC3860502

[11] Monroe AK, Lau B, Mugavero MJ, Mathews WC, Mayer KH, Napravnik S, et al Heavy Alcohol Use Is Associated With Worse Retention in HIV Care. J Acquir Immune Defic Syndr. 2016;73(4):419–25. Epub 2016/10/30. 10.1097/QAI.0000000000001083 27243904 PMC5085857

[12] Vagenas P, Azar MM, Copenhaver MM, Springer SA, Molina PE, Altice FL. The Impact of Alcohol Use and Related Disorders on the HIV Continuum of Care: a Systematic Review: Alcohol and the HIV Continuum of Care. Curr HIV/AIDS Rep. 2015;12(4):421–36. Epub 2015/09/29. 10.1007/s11904-015-0285-5 26412084 PMC4643391

[13] World Health Organization. Global status report on alcohol and health 2018. Geneva: World Health Organization, 2018.

[14] Egger M, Ekouevi DK, Williams C, Lyamuya RE, Mukumbi H, Braitstein P, et al Cohort Profile: The international epidemiological databases to evaluate AIDS (IeDEA) in sub-Saharan Africa. Int J Epidemiol. 2012;41(5):1256–64. 10.1093/ije/dyr080 21593078 PMC3465765

[15] World Health Organization. Interim WHO clinical staging of HIV/AIDS and HIV/AIDS case definitions for surveillance. Geneva: World Health Organization, 2005.

[16] World Health Organization. Guideline on when to start antiretroviral therapy and on pre-exposure prophylaxis for HIV. Geneva: World Health Organization, 2015. 26598776

[17] Babor TE, Higgins-Biddle J, Dauser D, Higgins P, Burleson JA. Alcohol screening and brief intervention in primary care settings: implementation models and predictors. J Stud Alcohol. 2005;66(3):361–8. Epub 2005/07/29. 10.15288/jsa.2005.66.361 . 16047525

[18] Babor TF, Higgins-Biddle JC, Higgins PS, Gassman RA, Gould BE. Training medical providers to conduct alcohol screening and brief interventions. Subst Abus. 2004;25(1):17–26. Epub 2004/06/18. 10.1300/J465v25n01_04 15201108 PMC3552328

[19] Babor TF, Higgins-Biddle JC, Saunders JB, Monteiro MG. AUDIT The Alcohol Use Disorders Identification Test Guidelines for Use in Primary Care, 2nd edition Geneva: World Health Organization; 2001.

[20] Saunders JB, Aasland OG, Babor TF, de la Fuente JR, Grant M. Development of the Alcohol Use Disorders Identification Test (AUDIT): WHO Collaborative Project on Early Detection of Persons with Harmful Alcohol Consumption—II. Addiction. 1993;88(6):791–804. Epub 1993/06/01. 10.1111/j.1360-0443.1993.tb02093.x . 8329970

[21] Papas RK, Sidle JE, Wamalwa ES, Okumu TO, Bryant KL, Goulet JL, et al Estimating alcohol content of traditional brew in Western Kenya using culturally relevant methods: the case for cost over volume. AIDS Behav. 2010;14(4):836–44. Epub 2008/11/19. 10.1007/s10461-008-9492-z 19015972 PMC2909349

[22] Geng EH, Glidden DV, Bwana MB, Musinguzi N, Emenyonu N, Muyindike W, et al Retention in care and connection to care among HIV-infected patients on antiretroviral therapy in Africa: estimation via a sampling-based approach. PLoS One. 2011;6(7):e21797 Epub 2011/08/06. 10.1371/journal.pone.0021797 21818265 PMC3144217

[23] Geng EH, Glidden DV, Emenyonu N, Musinguzi N, Bwana MB, Neilands TB, et al Tracking a sample of patients lost to follow-up has a major impact on understanding determinants of survival in HIV-infected patients on antiretroviral therapy in Africa. Trop Med Int Health. 2010;15 Suppl 1:63–9. Epub 2010/07/14. 10.1111/j.1365-3156.2010.02507.x 20586962 PMC3038920

[24] Vittinghoff E, editor. Regression methods in biostatistics: linear, logistic, survival, and repeated measures models. 2nd ed New York: Springer; 2012.

[25] Austin PC, Fine JP. Practical recommendations for reporting Fine-Gray model analyses for competing risk data. Stat Med. 2017;36(27):4391–400. Epub 2017/09/16. 10.1002/sim.7501 28913837 PMC5698744

[26] Kleinbaum DG, Klein DJ. Survival analysis: a self-learning text. 3rd ed New York, NY: Springer; 2012.

[27] Santos GM, Emenyonu NI, Bajunirwe F, Rain Mocello A, Martin JN, Vittinghoff E, et al Self-reported alcohol abstinence associated with ART initiation among HIV-infected persons in rural Uganda. Drug Alcohol Depend. 2014;134:151–7. Epub 2013/10/31. 10.1016/j.drugalcdep.2013.09.025 24169501 PMC3885244

[28] Baum MK, Rafie C, Lai S, Sales S, Page JB, Campa A. Alcohol use accelerates HIV disease progression. AIDS Res Hum Retroviruses. 2010;26(5):511–8. Epub 2010/05/12. 10.1089/aid.2009.0211 20455765 PMC2875959

[29] Kowalski S, Colantuoni E, Lau B, Keruly J, McCaul ME, Hutton HE, et al Alcohol consumption and CD4 T-cell count response among persons initiating antiretroviral therapy. J Acquir Immune Defic Syndr. 2012;61(4):455–61. Epub 2012/09/08. 10.1097/QAI.0b013e3182712d39 22955054 PMC3541505

[30] Malbergier A, Amaral RA, Cardoso LD. Alcohol dependence and CD4 cell count: is there a relationship? AIDS Care. 2015;27(1):54–8. Epub 2014/09/03. 10.1080/09540121.2014.947235 . 25179410

[31] Samet JH, Cheng DM, Libman H, Nunes DP, Alperen JK, Saitz R. Alcohol consumption and HIV disease progression. J Acquir Immune Defic Syndr. 2007;46(2):194–9. Epub 2007/08/02. 10.1097/QAI.0b013e318142aabb 17667330 PMC2247363

