# Supplementary material for: Lower rates of ART initiation and decreased retention among ART-naïve patients who consume alcohol enrolling in HIV care and treatment programs in Kenya and Uganda
Source: PLoS One. 2020 Oct 23;15(10):e0240654. doi: 10.1371/journal.pone.0240654 (PMC7584184; doi:10.1371/journal.pone.0240654)
Supplement: Attachment — Submitted filename: Review comments.docx [file pone.0240654.s001.docx]

In this manuscript, the authors assessed alcohol use among 765 ART-naive HIV+ adults enrolling into care using AUDIT to classify drinkers as drinkers/non-drinkers, hazardous/non-hazardous drinkers, and hyper/non-hyper drinkers. They then compared the effects of various levels of alcohol use on retention in care, ART initiation delay, and death. They report findings that drinkers were less likely to be retained in care and more likely to delay ART initiation. Interestingly, these associations were only significant when comparing any alcohol use to no alcohol use but were not found among hazardous and hyper alcohol use levels. Significant limitations of the manuscript include clear definitions of the primary outcomes, a clear (and apparently short) time frame to assess the primary outcomes, and a small number of primary outcomes limiting the ability to detect statistical significance among the smaller strata of hazardous and hyper drinkers.

In general, revision of this paper would benefit from a more robust, nuanced review of the previous literature, clarification and re-organization of the study methods, and additional discussion in interpretation of the results. This research adds to the literature in understanding the more nuanced effects of levels of alcohol use on HIV care outcomes, aiding in the potential to target interventions to improve HIV care outcomes among the particularly high-risk group of HIV-positive drinkers.

Specific comments are as follows (line numbers not included on manuscript):

**TITLE**:

1. The title as written is vague. Suggest clarifying the intended meaning of “impact” in the title, e.g. “Prevalence of Alcohol Use and Impact on HIV Care Outcomes in ART-naïve Adults Enrolling in HIV Care in East Africa”

**ABSTRACT**

1. Methods: Would be more specific than “East Africa” as this affects interpretation of alcohol use prevalence; recommend specifying Kenya & Uganda
2. Methods: Because primary outcomes are measured over time, please include the duration of follow-up
3. Methods: Include the comparison groups for each classification, i.e. non-drinkers for all? Or non-hazardous, non-hyper, etc.?
4. Results: Suggest clarifying “any alcohol use was associated..” as hazardous and hyper use were not found to be associated with retention or ART outcomes.
5. Results: Given mortality is a primary outcome of the study, results regarding the association should be included within the abstract
6. Conclusions: The phrasing “significant delays in ART initiation” implies a period, however this is not provided in the results. I.e. is a significant delay 1 week or 1 year?

**INTRODUCTION**

1. 2^nd^ paragraph: “impact on initiation of ART and retention in care yet to be thoroughly examined” is perhaps an oversimplification of the literature. Certainly, there Is a gap, but a more nuanced discussion of the data would help to contextualize these findings.
2. Mention of "few EBM interventions” distracts from the point of the paragraph; suggest deleting
3. In general, the points raised here don’t connect to the study being done: i.e. the study does not introduce new measure of alcohol exposure, new measurement of retention, or adjust for more confounders than past studies

**METHODS**

1. In general, the ordering of this section is confusing. Recommend that the opening sentence include not only the study design type, but also the study period, study purpose and setting. I.e. “From January 2013 to June 2014, we conducted a prospective observational study to determine X among adults entering HIV care in Uganda and Kenya.” This will clarify and contextualize the subsequent detailed methods section. Furthermore, the subheading “Study Population” should be divided to include a section on “Measures.” Several portions of the text included under “Statistical methods” are descriptions of measures, rather than statistical methods and should be re-organized appropriately.
2. **Study Design/Ethics:** Was referral for alcohol counseling or treatment offered to hazardous and hyper drinkers?
3. **Study setting and SOC:** The study period is mentioned but not defined until later in the manuscript; please add study years with respect to ART eligibility criteria to aid interpretation of the SOC being described
4. **Study setting and SOC:** Both Kenya and Uganda updated ART guidelines to expand treatment to all individuals with a CD4<500 in 2014. Was this implemented during the study time period? Were all individuals started on ART based on a cut off of 350 (i.e. were pregnant women or those with a high CD4 and OIs excluded?)
5. **Study setting and SOC:** Please clarify ART eligibility criteria further (1) how were pregnant women and (2) those with OIs treated if CD4 > threshold?
6. **Study population:** Recommend a sub-heading of “Measures” after describing and participants and before describing AUDIT
7. **Study population:** Please provide reference for AUDIT validation in sub-Saharan African setting
8. **Study population:** You reference the “AUDIT version utilized”: was this not the standard AUDIT tool? If non-standard, please include in the supplemental information. The standardization of procedures used to assess alcohol use are unclear. If additional or supplemental questions were asked and conflicted with answers to the standard 10-question screen, which answer was used in the analysis? While standard to include a chart of types of alcohol and volume to aid classification of alcohol use, additional questions about frequency are not typically standard.
9. **Statistical methods:** The definition of “retention in care” is unclear. Firstly, “retention in care,” as defined, seems to measure “retention in care failure” as it is “patients actually disengaged from care. Second, earlier in the manuscript, it is stated that a normal interval between appointments is up to 6 months; however, retention in care failure is being assessed at 2 months following the last (non-missed?) appointment.
10. **Statistical methods:** The sentence “IN this manner, we were able to estimate” is editorial and does not describe methodology. Suggest deleting.
11. **Statistical methods:** For those who were consistently retained in care (i.e. did not require tracing), how was censoring done?
12. **Statistical methods:** How was the mortality outcome ascertained? Were death registers utilized?
13. **Statistical methods:** Please provide a clear definition of the 3^rd^ primary outcome, time from enrollment to ART initiation. This is later referred to as delays in ART initiation: please clearly define the outcome and use consistent terminology. Is ART initiation restricted based on ART eligibility by CD4 measured at the relevant visit?
14. **Statistical methods:** For all primary outcomes, what was the timeline from enrollment to outcome measurement? This is critical for interpretation of the outcomes; retention in care over 3 months is vastly different than over 2 years, for example.
15. **Predictors used in the analyses:** For the alcohol use categories, were non-drinkers (AUDIT=0) included in the groups non-hazardous drinkers? In non-hyper drinkers?
16. **Predictors used in the analyses** Regarding the terminology “hyper-drinkers”, is this used elsewhere in the literature? If yes, please provide a reference. If not, what was the rationale for combining standard categories, given mention in the introduction that non-standard definitions of alcohol use limit comparison across studies?

**Results**:

1. **Participant characteristics**: How many patients were screened and excluded based on the criteria outlined in methods? Distribution of reasons for exclusion?
2. What percent met eligibility for ART at enrollment? At subsequent visits? What was the mean follow up time and number of visits for participants?
3. “Initially 25% lost”: When is initially? Is this the follow up interval?
4. How was death ascertained? 7% of the 190 lost to program died (N=13?); how were the remaining 15 deaths determined?

**Discussion**

1. In general, the null findings for hazardous and hyper-drinkers seem most like attributable to a small sample size with a rare outcome that is unable to detect significance. The discussion should include attention to this possibility.
2. A more robust discussion of the studies limitations, including the assumptions of the statistical model, small sample size, observational nature/incomplete data, etc., is merited.

**Conclusion**:

1. The conclusion is over-stated. The study did not find that all amounts of alcohol use were association with lower rates of ART initiation and lower retention in care.
2. The 3^rd^ component of 90-90-90 is viral suppression, not retention in care; furthermore, retention in care is an intermediary step preceding suppression, but does not serve as a proxy for viral suppression
3. The conclusion that “its evaluation appears to be a significant target for intervention is overstated; however fair to conclude that screening for alcohol use in ART naïve patients enrolling in care may be able to identify patients at higher risk for delaying ART start despite eligibility and not being retained in care .

**Table 2:**

1. Change in the ordering of columns adds confusion. Please move binary drinkers to the first column to compare with Tables 1 & 3.
