# Supplementary material for: Lower rates of ART initiation and decreased retention among ART-naïve patients who consume alcohol enrolling in HIV care and treatment programs in Kenya and Uganda
Source: PLoS One. 2020 Oct 23;15(10):e0240654. doi: 10.1371/journal.pone.0240654 (PMC7584184; doi:10.1371/journal.pone.0240654)
Supplement: Attachment — Submitted filename: PONE-D-19-24950-2r-response-to-reviewers.docx [file pone.0240654.s003.docx]

**Reviewer # 2**

***METHODS***

***Study setting and standard of care***
*Paragraph two: ......Individuals enrolling in care have a CD4 count drawn ...... : could this be rephrased to read "Individuals enrolling in care have blood drawn for CD4 cell count "?*

Done

***Study procedures***
*a) The Alcohol Use Disorder Screening Test (AUDIT) ......> Change word "screening" to "Identification"*

Done

*b) Please provide the period of recall for alcohol use in this section - 1 yr?*

The term “over the past one year” was added to the sentence “The Alcohol Use Disorder Identification Test (AUDIT) questionnaire, which has been validated in a number of primary care medical settings, was used by a trained research assistant to collect patient’s alcohol use data **over the past one year**.”

***Statistical analyses***
*Paragraph two ......different dichotomous level of alcohol consumption (i.e., binary, hazardous and hyper drinking, the exposure)..... "Binary " changed to word "drinkers" or "any drinking"*

We have removed all accurate but, admittedly, awkward terms like “binary” or “dichotomous” drinking in favor of more colloquial terminology like “any alcohol use”. For example, the above paragraph was changed to “We performed three separate analyses, each investigating the possible effect of **different levels of alcohol consumption** (i.e., **any alcohol use**, hazardous and hyper drinking, the exposure) on the likelihood (sub-distribution hazard) of each of the three events of interest (ART initiation, death, and non-retention in care).” (changes bolded).

***Results***

*Competing risk analysis*

*Given the low rates of non-retention (10.6%, Table 2), this is approximately equal 76.6% higher odds or risk of non-retention ... [i]mprove sentence to read 77%( similar visually to the aSHR 1.77) instead 76.6%*

Done

*Review the use of the words " higher odds or risk". The author's reference 26 [Practical recommendations for reporting Fine‐Gray model analyses for competing risk data] recommends that the SHR is best reported as a rate rather that risk since the aSHR does not in and of itself quantify the magnitude of the effect of "any drinking" on the Cumulative incidence function of non retention.*

Done. For example, the above sentence now reads “Given the low rates of non-retention (10.6%, Table 2), this is approximately equal 77% higher **rate** of non-retention. Other alcohol exposure classifications (i.e., hazardous versus non-hazardous…” (change bolded).

***Discussion***
 *Sentence 1 . This study found a strong association between alcohol consumption and non-retention in
HIV care. - Suggestion to rephrase as "This study found a strong association between alcohol consumption within the last *** insert recall period) and non-retention in HIV care.*

Done with a reference to the one-year recall applied to the one-year recall

*Consequently, with less than 4% of study subjects dying during the study, there was very small power to detect any potential effects of alcohol consumption on mortality. Remove wording very small power and could possibly rephrase to " Consequently, with less than 4% of study subjects dying, our study was not powered to detect any potential effects of alcohol consumption on mortality.*

Done

*Rephrase sentence " In addition, dichotomizing the data in*

*Combined, these limitations in our data collection may account for finding an association
of alcohol consumption with decreased retention in care and delays in ART initiation, but not
confirming the same findings among those at the highest alcohol consumption classifications."*

Done
